# Machine learning integration for predicting the effect of single amino acid substitutions on protein stability

**DOI:** 10.1186/1472-6807-9-66

**Published:** 2009-10-19

**Authors:** Ayşegül Özen, Mehmet Gönen, Ethem Alpaydın, Türkan Haliloğlu

**Affiliations:** 1 Department of Chemical Engineering, Polymer Research Center, Boğaziçi University, İstanbul, Turkey; 2 Department of Computer Engineering, Boğaziçi University, İstanbul, Turkey

## Abstract

**Background:**

Computational prediction of protein stability change due to single-site amino acid substitutions is of interest in protein design and analysis. We consider the following four ways to improve the performance of the currently available predictors: (1) We include additional sequence- and structure-based features, namely, the amino acid substitution likelihoods, the equilibrium fluctuations of the alpha- and beta-carbon atoms, and the packing density. (2) By implementing different machine learning integration approaches, we combine information from different features or representations. (3) We compare classification vs. regression methods to predict the sign vs. the output of stability change. (4) We allow a reject option for doubtful cases where the risk of misclassification is high.

**Results:**

We investigate three different approaches: early, intermediate and late integration, which respectively combine features, kernels over feature subsets, and decisions. We perform simulations on two data sets: (1) S1615 is used in previous studies, (2) S2783 is the updated version (as of July 2, 2009) extracted also from ProTherm. For S1615 data set, our highest accuracy using both sequence and structure information is 0.842 on cross-validation and 0.904 on testing using early integration. Newly added features, namely, local compositional packing and the mobility extent of the mutated residues, improve accuracy significantly with intermediate integration. For S2783 data set, we also train regression methods to estimate not only the sign but also the amount of stability change and apply risk-based classification to reject when the learner has low confidence and the loss of misclassification is high. The highest accuracy is 0.835 on cross-validation and 0.832 on testing using only sequence information. The percentage of false positives can be decreased to less than 0.005 by rejecting 10 per cent using late integration.

**Conclusion:**

We find that in both early and late integration, combining inputs or decisions is useful in increasing accuracy. Intermediate integration allows assessing the contributions of individual features by looking at the assigned weights. Overall accuracy of regression is not better than that of classification but it has less false positives, especially when combined with the reject option. The server for stability prediction for three integration approaches and the data sets are available at .

## Background

In protein design and analysis, understanding the stability in sequence, structure, and function paradigms is of importance [1] and hence there is a need for predicting the protein stability change due to mutation. Single amino acid mutations can significantly change the stability of a protein structure [2]. To acquire a set of experimental annotations for every possible random mutation is combinatorial and requires significant resources and time. Thus, accurate computational prediction would be of use for suggesting the destructive mutations as well as the most favorable and stable novel protein sequences. To this end, the prediction of protein stability change due to amino acid substitutions remains a challenging task in the field of molecular biology.

Recent approaches fall into two major types: energy-based methods and machine learning approaches. Energy-based methods using physical, statistical, or empirical forcefields perform a direct computation of the magnitude of the relative change in the free energy [3-8]. Average assignment method [7] and different machine learning algorithms, such as support vector machines [2], neural networks [9], and decision trees [7] are trained on a data set to predict protein stability change. There are also hybrid approaches that combine energy-based and machine learning methods [10-12]; they basically generate the input features fed into machine learning algorithms using energy-based models.

One can predict the direction towards which the mutation shifts the stability of the protein (namely the sign of ΔΔ*G*). It could be positive or negative, corresponding to an increase or decrease in stability, respectively. From a machine learning perspective, this is a binary classification task, where given ***x***, information about the single-site amino acid substitution, the aim is to decide whether this is a positive or negative example, depending on whether the mutation is favorable or not. A third class of "doubt" can be defined for small changes that may be considered insignificant, and in such a case, one can train a three-class classifier [13] or a two-class classifier with the reject option.

Given a sample of *n *independent and identically distributed training instances, (***x***_**1**_, *y*_1_),(***x***_**2**_, *y*_2_), ...,(***x***_***n***_, *y*_*n*_), where ***x***_***i ***_is the *d*-dimensional input vector coding the relevant information and *y*_*i *_∈ {-1, +1} is its class label, *i *= 1, ..., *n*, a classifier estimates *P*(+|***x***) and assigns the test instance to the positive class if *P*(+|***x***) > 0.5, and to the negative class otherwise. There can be different representations in coding ***x***. Deciding on the best data representation used is as important as selecting the classification algorithm.

Another possibility in solving this using machine learning is to define it as a regression problem with ΔΔ*G *directly as the numeric output. One can then decide based on whether the prediction is positive or negative, and again predictions that are close to zero can be rejected if the risk of misclassification is high. No single machine learning algorithm nor representation, in classification or regression, induces always the most accurate learner in any domain. The usual approach is to try many and choose the one that performs the best on a separate validation set unused during training. Recently, it has been shown that accuracy may be improved by combining multiple learners [14,15]. There are three possible methods for combining multiple learners: early, late, and intermediate integration [16].

In early integration, inputs are concatenated as one large vector and a single learner (classifier or regressor) is used. In late integration, multiple classifiers/regressors are trained over different inputs and their decisions are combined by a trained learner. These two approaches can be applied with any classification/regression algorithm.

Late integration has been extensively used in bioinformatics. Weighted voting was used in classifier combination for protein fold recognition [17]. Majority voting was used for prediction of the drug resistance of HIV protease mutants [18], secondary structure prediction [19], detecting rare events in human genomic DNA [20] and identification of new tumor classes using gene expression profiles [21]. A trained combiner was used for secondary structure prediction [22,23]. A mixture of localized experts was used for gene identification [24]. Cascading, which is a multi-stage sequential combination method, was used for secondary structure prediction [25].

Support vector machines allow combination in a third way, using multiple kernels; this is also called intermediate integration [16]. Kernel functions basically measure similarity between data instances and a single learner can combine separate kernels for different data sources, instead of combining data before training a single learner (as in early integration) or combining decisions from multiple learners (as in late integration).

Intermediate integration was used for protein location prediction and protein function prediction tasks, respectively, by combining kernels applied to different representations such as protein sequences, hydropathy profile, protein interactions, and gene expressions [26,27]. This method is also used in glycan classification by combining different tree kernels [28].

Our work has four aspects: (1) Introduction of new protein residue features: The temperature factors of the backbone and side-chain carbon atoms (*B-factor*) that reflect the thermal mobility/flexibility of the mutated residue; the local packing information in a higher resolution than that has previously been incorporated by considering the side-chain atoms as well; amino acid substitution likelihoods from PAM250 matrix. (2) Implementation of three different machine learning approaches (early, late, and intermediate integration), two of which, namely late and intermediate, have not been used before in the computational prediction of protein stability change. (3) Comparison of classification and regression methods. (4) The use of a reject option in both classification and regression to check for cases where the learner has low confidence.

## Data

### Data Sets

The first data set (S1615) was compiled from the data available online [29], originally extracted [9] from the ProTherm database [30]. This data set has been used previously and provides a basis for comparison [2,9,31]. The set originally contains 1615 single-site mutation data from 42 different proteins. Each instance has the following features: PDB code of the protein, mutated position and mutation itself, solvent accessibility, pH value, temperature (T), and the change in the free energy, ΔΔ*G*, due to a mutation in a single position. As there are instances for the same mutation and position where ΔΔ*G *differs with T and pH values, T and pH are kept as features in our data set. A subset (388 instances) of the training set (1615 instances) was previously used as a test set for comparison between different predictors [2]. Though some studies include the test set also in the training set, we remove it from the training set to have disjoint training and test sets, as done in [2].

We also extract an up-to-date version (as of July 2, 2009) (S2783) that contains 2783 single-site mutations with known PDB code of the protein and ΔΔ*G *values also from the ProTherm database. On this larger data set, we implement and compare both classification and regression integration methods and also their versions with the reject option.

### Added Features

The substitution frequency of an amino acid for another is considered here as an additional feature with the Point Accepted Mutation (PAM) matrix [32]. PAM250 is chosen for the score of each amino acid substitution and is based on the frequency of that substitution in closely related proteins that have experienced a certain amount of evolutionary divergence.

Another feature considered is the mobility/flexibility of the amino acid position in a given structure. The *B-factor*s reported in the PDB file is a good and quick indicator of this feature. Neighbors of the mutated residue in both amino acid sequence and 3D structure are the two other features that have been used recently [2,9]. A window size of seven in the sequence [2] and a cutoff distance of 9Å in space was previously used to find the neighbors of the mutated position as the optimum sequence length and distance, respectively [9]. In our implementation, in addition to alpha-carbon atoms (*C*_*α*_), beta-carbon (*C*_*β*_) atoms are also considered to reflect the packing at a relatively higher resolution.

A mutation in a position of a protein sequence will change the number of side-chain atoms of the residue in that position. This may trigger a conformational change or local readjustments that may result also in a change in the atomic packing around that residue and the fluctuations of the surrounding residues and the mutated residue itself. Nevertheless, as in other studies [2,9,31], we neglect this effect.

Removing the instances with non-available features and the redundant instances from S1615 leaves us with training and test sets of 1122 and 383 instances with total of 31 and 14 proteins. Stabilizing mutations are 32.35 per cent and 11.49 per cent, respectively. After removing the instances with non-available features, S2783 reduces to 2471 instances from 68 different proteins and 755 of them (30.55 per cent) are stabilizing mutations. Both data sets are available online.

Table 1 gives a list of the representations, original features, and the new features that we introduce. The information coming only from the sequence (SO), and the topology of the protein structure (TO), and both (ST) are encoded in the same way as defined in previous studies [2]. An added asterisk, for example, (SO*), denotes the representation with newly added features. Neighbors of the mutated position in the sequence, mutation, T, and pH are encoded in SO/SO*. Sequence information is not used in TO/TO*; instead, spatial neighbors and the solvent accessibility of the mutated position are encoded. In ST/ST*, all information are combined. The substitution likelihood of an amino acid is added to the existing data as a new feature in all three representations. Crystallographic *B-factor*s of the *C*_*α *_and *C*_*β *_atoms are used in TO* and ST*. For discrete features like amino acid identities, *1-of-n encoding *is used, that is, if the variable can take one of *n *different values, one is set to 1 and all others to 0.

**Table 1:** Representations, original features, and the new features.

**Repr.**	**Original Feat.**	**New Repr.**	**New Feat.**
SO	± 3 neighbors (± 3 NE) Mutation (MUT) T/pH	SO*	PAM250 (PAM)

TO	Mutation (MUT) *C*_*α *_contacts (CA) SA/T/pH	TO*	PAM250 (PAM) *C*_*α *_B-factor (BFA) *C*_*α *_B-factor (BFB) *C*_*α *_and *C*_*β *_contacts (CB)

ST	± 3 neighbors (± 3 NE) Mutation (MUT) *C*_*α *_contacts (CA) SA/T/pH	ST*	PAM250 (PAM) *C*_*α *_B-factor (BFA) *C*_*α *_B-factor (BFB) *C*_*α *_and *C*_*β *_contacts (CB)

In all three representations, amino acid substitution likelihood is used as a feature. *B-factors* of the C_*α*_ and C_*β *_atoms and spatial neighbor determined using both C_*α *_ and C_*β *_atoms are features introduced into TO and ST. The abbreviations are given only for the features that we add.

## Methods

### The Effect of Adding New Features to the Original Data Sets

To each of the three representations (SO, TO, or ST), the new features are added one at a time and as combinations of two and three (see Table 2). Since all the new features except PAM are structure-related, they are not added to SO. All of the new features, including PAM, are added to both TO and ST. We end up with (2^1 ^(PAM) for SO and 2^4 ^(PAM, CB, BFA, BFB) for each of TO and ST combinations) a total of 34 possible feature sets (all of which include the mutation, T, and pH information).

**Table 2:** The list of 34 possible input feature sets.

**#**	**Representation**	**PAM**	**CB**	**BFA**	**BFB**
1	SO	-	-	-	-
2	SO	+	-	-	-

3	TO	-	-	-	-
4	TO	+	-	-	-
5	TO	-	+	-	-
6	TO	-	-	+	-
7	TO	-	-	-	+
8	TO	+	+	-	-
9	TO	+	-	+	-
10	TO	+	-	-	+
11	TO	-	+	+	-
12	TO	-	+	-	+
13	TO	-	-	+	+
14	TO	+	+	+	-
15	TO	+	+	-	+
16	TO	+	-	+	+
17	TO	-	+	+	+
18	TO	+	+	+	+

19	ST	-	-	-	-
20	ST	+	-	-	-
21	ST	-	+	-	-
22	ST	-	-	+	-
23	ST	-	-	-	+
24	ST	+	+	-	-
25	ST	+	-	+	-
26	ST	+	-	-	+
27	ST	-	+	+	-
28	ST	-	+	-	+
29	ST	-	-	+	+
30	ST	+	+	+	-
31	ST	+	+	-	+
32	ST	+	-	+	+
33	ST	-	+	+	+
34	ST	+	+	+	+

The new features to each of the three representations (SO, TO or ST) are added one at a time and as combinations of two and more. The original features are already given in Table 1 and are not shown here.

### Performance Assessment

Having already left 383 test instances out as the test set for S1615, we use 20-fold cross-validation (cv) on the 1122 training instances using 19/20 = 95 per cent for training proper and 5 per cent for validation. The best cross-validation strategy, that is, the number of folds, gets the best trade-off between the total amount of computation and training set size. With *k *folds, one needs *k *sets of training and validation and uses (*k *- 1)/*k *of data for training. We decided that the best is with *k *= 20; with higher *k *(or using jackknife), there is too much computation and with smaller *k*, training set gets small and variance increases. Classes should be represented in the right proportions when subsets of data are held out, not to disturb the class prior probabilities and we fulfill this requirement by stratification. Repeating training 20 times, we choose the hyperparameter set that has the highest average validation accuracy. The 20 classifiers trained on the 20 training folds for that hyperparameter set are tested on the test set. If we are required to perform classifier combination, we use the same training and validation sets also for the combiner due to the small size of the training set [33].

For all three integration methods, we use our own code; MOSEK [34] is used for solving the optimization problems of support vector machines. We report averages over 20 test results obtained by testing the trained classifier of each fold on the test set; for comparing classifiers, we use the paired *t*-test over these 20 results.

**Figure 1:**
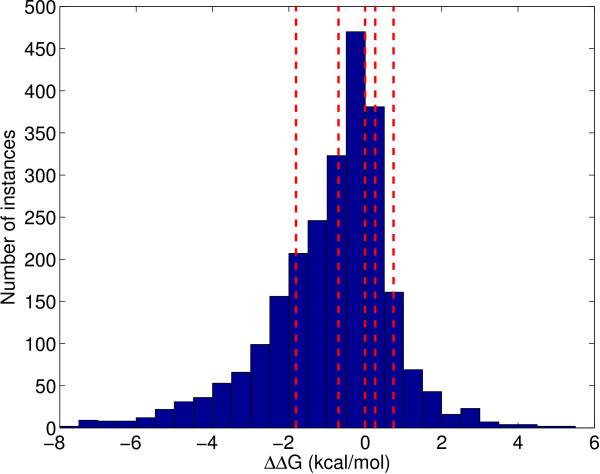
**Distribution of S2783 data over the free energy change due to single-site mutation, ΔΔ*G***. The regions separated by dashed lines are used to obtain similar training and test splits. Random one-third of the instances in each region is reserved for testing and the remaining two-third is used in training.

We use a slightly different methodology for S2783 because we train both classification and regression methods. First, we determine 3 split points for both stabilizing and destabilizing mutations as shown in Figure 1. Each split contains approximately the same number of data instances as the other two splits of the same class. This splitting mechanism both maintains stratification and ensures that we give the regressors training instances with diverse output values. Then, we take one-third of each split randomly to the test set and the remaining two-third is reserved as the training set. We apply 20-fold cv on the training set and obtain 20 folds. The learners (both classifiers and regressors) are trained on the 20 training folds and tested on the test set. The hyperparameter set that has the highest average validation accuracy for classification or the lowest mean square error for regression is selected and tested on the test set 20 times with the trained learners. This whole process is replicated 10 times each time using a different random test set. As a result, we obtain 10 × 20 test set results and report the average of these results.

The accuracies on the test set are calculated as given in Table 3 where *TP*, *FP*, *TN*, and *FN*, respectively refer to the number of true positives, false positives, true negatives, and false negatives. Precision, recall, and *FP *rate are evaluation measures which give information about the reliability of the predictor. The same measures are also reported for regression methods after converting the output of the regressor to a class prediction by looking at the sign.

**Table 3:** Performance evaluation measures.

Accuracy	
Error Rate	

Precision	

Recall	

FP Rate	

As we can see from Figure 1, ΔΔ*G *values are clustered around zero and small changes in the prediction of a learner may change the predicted label for a test instance. When the risk of misclassification is high, we can allow a predictor to give a reject decision. We define a risk matrix in Table 4 where *r *is the reject option, and the rows and columns correspond to the true and predicted class labels, respectively.

**Table 4:** Risk matrix.

		**Decision**		
		**+**	**-**	***r***
	
**Truth**	+	0	*λ*	1
	
	-	*αλ*	0	1

Predicting the class label correctly does not incur any cost at all. If the learner rejects, a unit cost incurs. If the learner makes a prediction error, it pays a misclassification cost *λ *for *FN *and *αλ *for *FP *where *α *is the trade-off parameter for *FP *and usually depends on the application. These misclassification costs should be larger than 1 in order to make the learner reject when it is not confident about its prediction. Given a risk matrix and *P*(+*|****x***), we can calculate the risks of three possible actions as follows:



and the best action is selected as the action with minimum risk. One can then solve for the rejection thresholds based on the values of *λ *and *α*. For example, if *λ *= 2 and *α *= 2, then we choose



We experiment with different *λ *(2, 5, 10) and *α *(1, 2, 5) values. If *α *= 1, this means that *FP *and *FN *have equal misclassification costs assigned to them. In our case, by taking *α *> 1, we say that predicting a destabilizing mutation as a stabilizing one is costlier than the other way around.

For regression where the output is not a probability but a number, we can not analytically solve for the two thresholds but need to do an exhaustive search. We search for two thresholds *θ*_1 _(< 0) and *θ*_2 _(> 0) on the validation sets given the values of *λ *and *α *that minimize the total classification risk. We choose the negative class if the regression output, *y*, for a specific test instance is less than *θ*_1_, reject if *θ*_1 _<* y *<*θ*_2_, and choose the positive class if *y *> *θ*_2_.

### Early Integration

Different classifiers make different assumptions about the data and may fail in different instances [14]. We train three classifiers, namely *k*-nearest neighbor estimator, decision tree, and support vector machine, using SO/SO*, TO/TO*, and ST/ST* representations. We use a single regression method, namely support vector regression, on all representations.

#### *k-Nearest Neighbor (k-NN) Classifier *

The *k*-NN classifier assigns the input to the class by taking a majority vote among its *k *neighbors. The best value of *k *is chosen from the set of 1, 3, 5, 7, 9, and 11 using 20-fold cv.

#### Decision Tree (DT)

A DT is a hierarchical model whereby the local region is identified in a sequence of recursive splits. When there is noise, growing the tree until it is purest, we may grow a very large tree. To alleviate such overfitting, tree construction ends when nodes contain few examples; this threshold, *τ*, is the hyperparameter to be tuned. *τ *parameter is selected from the trial values of 56 (5 per cent of the training set), 28, and 14 for S1615 (80, 40, and 20 for S2783).

#### Support Vector Machine (SVM)

SVM finds the linear discriminant in the feature space with the maximum margin [35]. SVM uses the training data in the form of dot products and allows embedding another feature space via kernel functions. The RBF (radial basis function) kernel was recently reported to work best for stability prediction [2]. The regularization parameter, *C*, is chosen from (0.01, 0.1, 1, 10, 100) and the kernel width, *γ*, is chosen from (0.25*r*, 0.5*r*, *r*, 2*r*, and 4*r*) where *r *is the average nearest neighbor distance over the training set.

#### Support Vector Regression (SVR)

SVR is an extension to SVM for regression problems [36]. The regularization parameter, *C*, is chosen from (0.01, 0.1, 1, 10, 100) and the width parameter of the RBF kernel, *γ*, is chosen from (0.25*r*, 0.5*r*, *r*, 2*r*, and 4*r*) where *r *is the average nearest neighbor distance over the training set, the regression tube width, ϵ, is selected from (0.05, 0.10, 0.15).

### Late Integration

It is possible to learn to combine the decisions of classifiers by a combiner classifier. By training the three classifiers described above with 34 data sets (see Table 2), we get 102 different combination triplets of (*R.D.B*) outputs where *R*, *D*, and *B *stand for *Representation*, *Data set*, and *Base-learner*. The output of the combiner is the best subset combination of these 102 triplets. The two criteria to select the best combination are accuracy and diversity, in that, we want (*R.D.B*) triplets that fail in different regions of the input space. In order to see to what extent any two classifiers are correlated, McNemar's test is used [15]. The same procedure can also be applied to combine regressors. We obtain 34 different regressors and the combiner chooses a subset from those. The correlation coefficient between the output of two regressors can be used to check the diversity between these regressors; a small correlation coefficient means that the two regressors are diverse.

The algorithm for selecting the most accurate and most diverse (*R.D.B*) triplets is a greedy, forward algorithm for subset selection. We start with an initial (*R.D.B*) that is the most accurate and search through the rest of the (*R.D.B*) triplets for those that are different from the initial one at significance level of *α *= 0.05 by McNemar's test. We add the most accurate one among those and iterate until there is no further improvement. The posterior probability outputs of the selected classifiers are then used to train a combiner that is an SVM with the linear kernel. The pseudocode of the algorithm is given in Table 5. The algorithm for combining regressors is very similar to Table 5 except three basic differences: (1) We select the regressor with the minimum mean squared error among candidate regressors. (2) We use correlation coefficient as the diversity measure between regressors. (3) We combine the outputs of selected regressors with a combiner that is an SVR with the linear kernel.

**Table 5:** The algorithm to select the classifiers to be combined.

1:	Initialize the subset *Z *as empty set
2:	Initialize the subset *R *as all possible 102 (*R.D.B*) groups
3:	Remove the most accurate (*R.D.B*) from *R *and add to *Z*
4:	Perform McNemar's test for all pairs between *Z *and *R*
5:	Decrease the degree of confidence, *α*, for McNemar's test
6:	**if **There is at least one diverse (*R.D.B*) in *R ***then**
7:	Select the most accurate and most diverse (*R.D.B*) from *R *and add it to *Z*
8:	Go to **Step 4**
9:	**else**
10:	Use the (*R.D.B*) triplets in *Z *as the current base-learners to be combined
11:	**end if**

The aim is to select the most accurate and at the same time the most diverse classifiers.

### Intermediate Integration

When using multiple kernels in support vector machines, there are two different possibilities [16]: We can calculate kernel functions on different representations or calculate different kernel functions on the same representation.

One can take a sum over different kernels and summation rule is applied successfully in computational biology [37] where heterogeneous data sets exist by the nature of the biological problems. 

Replacing the kernel function with a weighted summation of *p *kernel functions was proposed [38,39]:



where the combination weights (*η*_*m*_) are new parameters optimized in training. In addition to the flexibility of constructing weighted combination rules, using multikernel SVMs provides two important advantages: (1) Information can be extracted about the classification task at hand. The feature sets used in kernel functions with larger weights are understood to give more relevant information in terms of classification. For example, obtaining information about important features in biological problems such as disease diagnosis and drug development is as important as classification accuracy. (2) Kernel functions with zero weights can be eliminated. If such feature sets are obtained by using costly and time consuming experimental procedures, this decreases the overall complexity and cost.

For regression using intermediate integration, we use a variant of the localized multiple kernel learning model [40]. Kernel combination weights can be modeled by using the softmax function as follows:



where the softmax guarantees that *η*_*m *_≥ 0 and , and *u*_*m *_are the kernel-specific parameters we need to learn. These parameters are optimized during training in an iterative manner.

In intermediate integration, we combine RBF kernels over feature subsets that form SO/SO*, TO/TO*, and ST/ST*. Their width parameters are selected as the average nearest neighbor distances in the corresponding feature subsets.

## Results

### S1615 Data Set

#### Early Integration

We finetune the hyperparameters by inspecting the 20-fold cv misclassification error. For *k*-NN, *k *= 1 gives the most accurate cv results. The best parameter values for SVMs are (*C *= 100, *γ *= *r*), (*C *= 100, *γ *= *r*), and (*C *= 1, *γ *= *r*) for SO/SO*, TO/TO*, and ST/ST*, respectively. Decision tree parameter, *τ*, is validated to be 14 in all representations.

**Figure 2:**
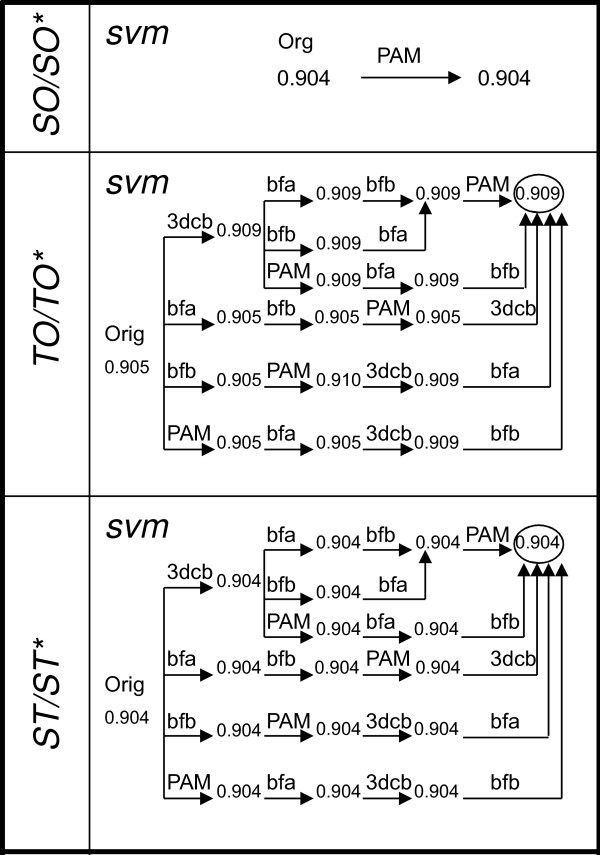
**Accuracy of the best (R.D.B) triplets in early integration for each representation of the S1615 data set**. Effect of adding each extra feature to the set of original features is observed by adding each, one at a time, and combinations of two or more. SVM is the best classifier for all representations.

Accuracies of the best *(R.D.B) *triplets for each representation of the data are given in Figure 2. The effect of adding each extra feature is observed by adding one at a time and in combinations of two or more. SVM yields the most accurate predictions in all three representations. The introduction of PAM into SO has no effect on accuracy, which is 0.904. The average testing accuracy for TO increases from 0.904 to 0.909 with the help of new features, which is not statistically significant. Our results show that adding extra features to ST does not improve the accuracy of 0.904. The best accuracies with original and extra features for early integration are given in Table 6. Table 7 lists the precision, recall, and *FP *rate values on the test set for the best classifiers for all three representations.

**Table 6:** Early integration results for S1615 data set.

	***k*-NN**		**DT**		**SVM**	
	**cv**	**test**	**cv**	**test**	**cv**	**test**
**SO**	0.814	0.778	0.752	0.703	0.838	0.904
SO*	0.812	0.781	0.766	0.702	0.839	0.904

**TO**	0.812	0.819	0.770	0.739	0.822	0.905
TO*	0.817	0.844	0.788	0.756	0.825	0.909

**ST**	0.814	0.777	0.771	0.734	0.838	0.904
ST*	0.817	0.775	0.800	0.729	0.842	0.904

The accuracy of each base-learner trained with original data and with extra features added in SO/SO*, TO/TO* or ST/ST*. The values reported for each classifier are respectively the validation and test accuracies of the original representation and the new representation.

**Table 7:** The precision, recall, and *FP *rates of the most accurate classifiers on the test set in early integration for S1615 data set.

	**SVM**		
	**SO**	TO*	ST*
**Precision**	0.711	0.800	0.702
**Recall**	0.284	0.282	0.284
***FP *rate**	0.015	0.009	0.016

#### Late Integration

For *k*-NN, we choose *k *= 5 to give more informative posterior probabilities, rather than 0/1 decisions, to the combiner in late integration.

The most accurate (*R.D.B*) triplet among all 102 classifier triplets is (ST.PAMCB.SVM) that denotes a support vector machine (*B*) trained with ST* (*R*) with the additional new features, PAM and packing density from *C*_*α *_and *C*_*β *_(D). The best complements turn out to be (TO.BFA.SVM), (ST.CBBFB.DT), and (TO.CBBFABFB.*k*-NN) using the selection method of Table 5. When the outputs of the *(R.D.B) *triplets are given to the SVM Combiner, the average accuracy is 0.903 on the test set and 0.847 on the validation set (see Table 8). This accuracy is comparable to the reported values in previous studies [2,7,31]. Similarities between selected *(R.D.B) *triplets calculated by McNemar's test are given in Table 9.

**Table 8:** Performance of late integration of the four triplets (ST.PAMCB.SVM), (TO.BFA.SVM), (ST.CBBFB.DT), and (TO.CBBFABFB.k-NN) for S1615 data set.

	**cv**	**test**
**Accuracy**	0.847	0.903
**Precision**	0.819	0.694
**Recall**	0.677	0.284
***FP *rate**	0.071	0.017

**Table 9:** McNemar's test results for the triplets (ST.PAMCB.SVM), (TO.BFA.SVM), (ST.CBBFB.DT), and (TO.CBBFABFB.*k*-NN) for S1615 data set.

	**(2)**	**(3)**	**(4)**
**(1) ST.PAMCB.SVM**	11.72	66.61	154.10
**(2) TO.BFA.SVM**		42.12	135.64
**(3) ST.CBBFB.DT**			41.32
**(4) TO.CBBFABFB.*k*-NN**			

#### Intermediate Integration

The test results for all data representations are given in Table 10. We can see that adding PAM to SO does not change the accuracy because PAM is assigned zero weight; but adding extra features to TO and ST increase the average accuracy by 4.6 per cent and 6.0 per cent, respectively; both improvements are statistically significant. The highest accuracy is obtained with TO* (0.879), which however is significantly less than 0.909 of early integration.

**Table 10:** Multikernel SVM test results as intermediate integration for S1615 data set.

	**Accuracy**	**Precision**	**Recall**	***FP *Rate**
**SO**	0.872	0.381	0.176	0.038
SO*	0.872	0.381	0.176	0.038

**TO**	0.833	0.343	0.485	0.122
TO*	0.879	0.459	0.258	0.040

**ST**	0.818	0.311	0.470	0.137
ST*	0.878	0.448	0.252	0.041

The kernel weights can be used to assess the relative importance of features (see Table 11). In all three representations, each feature subset except PAM has a combination weight (*η*_*m*_) greater than zero. The original representations have meaningful features for classification. The weights also show that ± 3 neighbors in the sequence carry as much information as ± 1 and ± 2 neighbors. In the modified representations (SO*, TO*, and ST*), the new weights indicate that the added features, except PAM, carry information for the stability of a protein. Local spatial composition with *C*_*α *_and *C*_*β *_(CB) has larger weight than *C*_*α*_ (CA), which highlights the contribution of side-chain packing to stability. Also, the information that reflects the extent of mobility/flexibility of each *C*_*α *_(BFA) and *C*_*β *_(BFB) have nonzero weights, implying that they are informative.

**Table 11:** The combination weights obtained for the original and modified features for S1615 data set.

**SO**	(0.19)1NE + (0.15)2NE + (0.31)3NE + (0.30)MUT + (0.03)T + (0.03)pH
SO*	(0.19)1NE + (0.15)2NE + (0.31)3NE + (0.30)MUT + (0.03)T + (0.03)pH + (0.00)PAM

**TO**	(0.36)MUT + (0.40)CA + (0.15)SA + (0.04)T + (0.04)pH

TO*	(0.21)MUT + (0.21)CA + (0.10)SA + (0.01)T + (0.01)pH + (0.00)PAM + (0.30)CB + (0.09)BFA + (0.07)BFB

**ST**	(0.04)1NE + (0.04)2NE + (0.09)3NE + (0.38)MUT + (0.25)CA + (0.11)SA + (0.04)T + (0.04)pH

ST*	(0.03)1NE + (0.02)2NE + (0.06)3NE + (0.20)MUT + (0.18)CA + (0.09)SA + (0.01)T + (0.01)pH + (0.00)PAM + (0.26)CB + (0.08)BFA + (0.06)BFB

#### Overall Comparison of Integration Methods

To be able to compare the three integration methods, in all three representations, we chose the version that has the highest average validation accuracy and compared the three. The ones chosen are given in Table 12 that shows the averages and standard deviations of validation and test accuracies. According to 20-fold paired *t*-test on the test results, there is no significant difference between early and late integration; both are significantly more accurate than intermediate integration.

**Table 12:** Comparison of best of three integration methods for S1615 data set.

	**early**	**late**	**intermediate**
	(ST.PAMCB.SVM)	(ST.PAMCB.SVM) + (TO.BFA.SVM) + (ST.CBBFB.DT) + (TO.CBBFABFB.*k*-NN)	(TO.PAMCBBFABFB.SVM)
**cv**	0.842 ± 0.047	0.847 ± 0.046	0.826 ± 0.044
**test**	0.904 ± 0.004	0.903 ± 0.005	0.879 ± 0.006

early = late > intermediate according to paired *t*-test

### S2783 Data Set

#### Early Integration

We finetune the hyperparameters by inspecting the 20-fold cv misclassification error and mean squared error for classifiers and regressors, respectively. For *k*-NN, *k *= 1 gives the most accurate cv results. Decision tree parameter, *τ*, is validated to be 20 in all representations. The best parameter values for SVMs are (*C *= 100, *γ *= *r*), (*C *= 10, *γ *= *r*), and (*C *= 100, *γ *= *r*) for SO/SO*, TO/TO*, and ST/ST*, respectively. (*C *= 10, *γ *= *r*) set works best for all SVR simulations but the tube width, ϵ, is selected as 0.05 or 0.10. The cv and test accuracies for each representation with different learners are given in Table 13. We see that SVM and SVR clearly outperform *k*-NN and DT by improving accuracy more than 1.5 per cent in all three representations. When we look at the effect of adding the new features to the original representations for SVM and SVR, we see that the new features do not change the test accuracy very much. The precision, recall, and *FP *rate values on the test set are also listed for SVM and SVR in Table 14, where we see that though SVM and SVR have comparable accuracies, SVR almost halves the *FP *rate, for example on ST*, it reduces from 0.078 to 0.040.

**Table 13:** Early integration results for S2783 data set.

	***k*-NN**		**DT**		**SVM**		**SVR**	
	**cv**	**test**	**cv**	**test**	**cv**	**test**	**cv**	**test**
**SO**	0.795	0.794	0.748	0.762	0.829	0.832	0.825	0.828
SO*	0.793	0.794	0.751	0.756	0.829	0.829	0.824	0.827

**TO**	0.804	0.803	0.762	0.769	0.821	0.824	0.813	0.818
TO*	0.806	0.799	0.770	0.780	0.826	0.829	0.818	0.824

**ST**	0.797	0.797	0.758	0.766	0.829	0.831	0.825	0.828
ST*	0.798	0.797	0.766	0.782	0.829	0.830	0.825	0.828

The accuracy of each base-learner trained with original data and with extra features added in SO/SO*, TO/TO* or ST/ST*. The values reported for each classifier and regressor are respectively the validation and test accuracies of the original representation and the new representation.

**Table 14:** The precision, recall, and *FP *rates of the most accurate classifiers and regressors on the test set in early integration for S2783 data set.

	**SVM**			**SVR**		
	**SO**	TO*	ST*	**SO**	TO*	ST*
**Precision**	0.790	0.807	0.784	0.854	0.868	0.855
**Recall**	0.612	0.579	0.614	0.527	0.501	0.529
***FP *rate**	0.072	0.061	0.075	0.040	0.034	0.040

#### Late Integration

First, 102 classifiers trained on S2873 data set are combined with the procedure explained in Table 5. We obtain the average accuracy as 0.832 on the test set and 0.830 on the validation set (see Table 15). Then, we combine 34 regressors trained, the average test set accuracy is 0.827 and the average validation accuracy is 0.819. Again, we see that in terms of accuracy, SVM and SVR are comparable, though the latter has higher precision and lower *FP *rate.

**Table 15:** Performance of late integration for S2783 data set.

	**SVM**		**SVR**	
	**cv**	**test**	**cv**	**Test**
**Accuracy**	0.830	0.832	0.819	0.827
**Precision**	0.795	0.790	0.853	0.858
**Recall**	0.604	0.615	0.495	0.520
***FP *rate**	0.071	0.073	0.038	0.038

#### Intermediate Integration

The test results for all data representations using multikernel SVM and SVR are given in Table 16. When we use multikernel SVM, we can see that adding extra features does not change accuracy. The highest accuracy is obtained with ST (0.806), which however is less than 0.832 of early integration. Using extra features in multikernel SVR does not help increase the accuracy either. The best accuracy performance is obtained with TO as 0.797.

**Table 16:** Multikernel SVM and SVR test results as intermediate integration for S2783 data set.

	**SVM**				**SVR**			
	**Accuracy**	**Precision**	**Recall**	***FP *Rate**	**Accuracy**	**Precision**	**Recall**	***FP *Rate**
**SO**	0.800	0.716	0.589	0.107	0.789	0.688	0.570	0.115
SO*	0.799	0.708	0.604	0.114	0.790	0.692	0.569	0.113

**TO**	0.805	0.710	0.621	0.113	0.797	0.705	0.580	0.107
TO*	0.802	0.697	0.629	0.122	0.792	0.677	0.611	0.129

**ST**	0.806	0.705	0.636	0.119	0.793	0.681	0.607	0.126
ST*	0.804	0.700	0.633	0.121	0.789	0.671	0.610	0.132

When we look at Tables 17 and 18, we can say that the added features carry information for predicting the energy change for single-site mutations even though they do not improve the average testing accuracy. As in S1615 data set, local spatial composition with *C*_*α *_and *C*_*β *_(CB) has larger weight than *C*_*α *_(CA) and the information that reflects the extent of mobility/flexibility of each *C*_*α *_(BFA) and *C*_*β *_(BFB) has nonzero weights.

**Table 17:** The combination weights obtained with SVM for the original and modified features for S2783 data set.

**SO**	(0.19)1NE + (0.20)2NE + (0.23)3NE + (0.27)MUT + (0.09)T + (0.03)pH
SO*	(0.19)1NE + (0.20)2NE + (0.22)3NE + (0.27)MUT + (0.09)T + (0.03)pH + (0.00)PAM

**TO**	(0.19)MUT + (0.56)CA + (0.17)SA + (0.05)T + (0.02)pH

TO*	(0.21)MUT + (0.23)CA + (0.12)SA + (0.06)T + (0.02)pH + (0.00)PAM + (0.23)CB + (0.07)BFA + (0.06)BFB

**ST**	(0.04)1NE + (0.03)2NE + (0.04)3NE + (0.21)MUT + (0.45)CA + (0.15)SA + (0.06)T + (0.02)pH

ST*	(0.02)1NE + (0.02)2NE + (0.03)3NE + (0.21)MUT + (0.21)CA + (0.11)SA + (0.06)T + (0.02)pH + (0.00)PAM + (0.19)CB + (0.06)BFA + (0.06)BFB

**Table 18:** The combination weights obtained with SVR for the original and modified features for S2783 data set.

**SO**	(0.15)1NE+ (0.25)2NE+ (0.22)3NE+ (0.31)MUT + (0.04)T + (0.02)pH
SO*	(0.16)1NE+ (0.26)2NE+ (0.22)3NE+ (0.29)MUT + (0.05)T + (0.01)pH + (0.01)PAM

**TO**	(0.25)MUT + (0.72)CA + (0.02)SA + (0.01)T + (0.00)pH

TO*	(0.28)MUT + (0.10)CA + (0.05)SA + (0.08)T + (0.03)pH + (0.01)PAM + (0.43)CB + (0.01)BFA + (0.01)BFB

**ST**	(0.02)1NE+ (0.02)2NE+ (0.02)3NE+ (0.26)MUT + (0.57)CA + (0.04)SA + (0.07)T + (0.03)pH

ST*	(0.01)1NE+ (0.01)2NE+ (0.01)3NE+ (0.30)MUT + (0.10)CA + (0.06)SA + (0.07)T + (0.01)pH + (0.00)PAM + (0.43)CB + (0.01)BFA + (0.01)BFB

#### Classification with Reject Option

**Figure 3:**
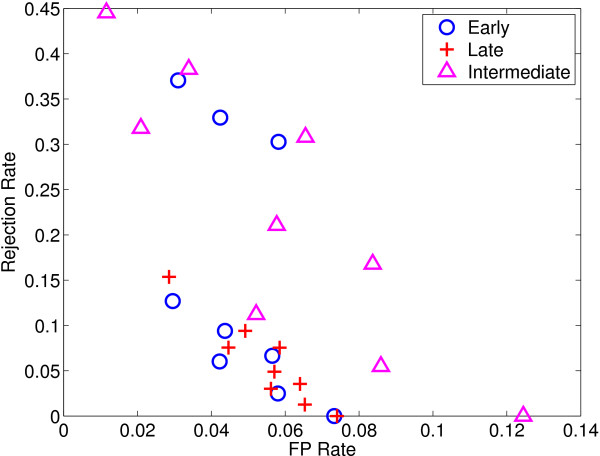
***FP *rate vs. rejection rate for each integration method using SVM as the base learner with changing *λ *and *α *values**.

**Figure 4:**
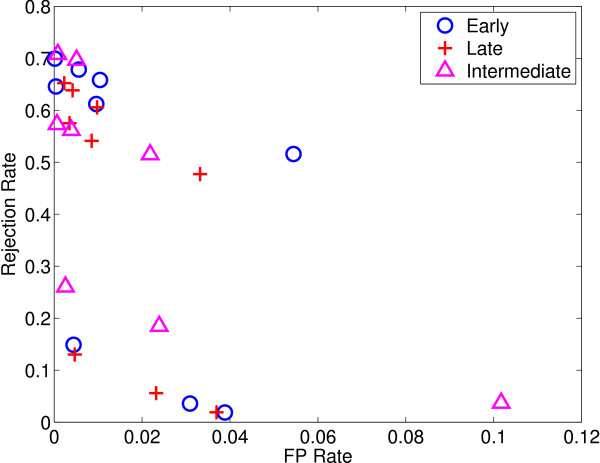
***FP *rate vs. rejection rate for each integration method using SVR as the base learner with changing *λ *and *α *values**.

We also perform simulations with reject option both for classification and regression, and give the performance measures obtained with early integration using SO (see Tables 19 and 20), late integration (see Tables 21 and 22), and intermediate integration using TO* (see Tables 23 and 24), respectively. We see that increasing *λ *and *α *values increases the accuracy of predictors and decreases *FP *rate at the cost of rejecting some instances. The selection of *λ *and *α *values is of crucial importance and depends on the loss incurred for making wrong decisions. Figures 3 and 4 show *FP *rate and rejection rate values for all integration approaches using SVM and SVR with the tried (*λ*, *α*) pairs. We see that using late integration for SVM case generally gives lower rejection rate than early and intermediate integration for a given *FP *rate; SVR can attain much lower *FP *rate but needs to reject more.

**Table 19:** Performance measures of SVM early integration (SO) for S2783 data set with reject option.

		**cv**					**test**				
***λ***	***α***	**Acc.**	**Prec.**	**Recall**	***FP *Rate**	**Reject**	**Acc.**	**Prec.**	**Recall**	***FP *Rate**	**Reject**
2	1	0.829	0.793	0.602	0.071	0.000	0.831	0.788	0.615	0.073	0.000
2	2	0.834	0.813	0.582	0.059	0.024	0.839	0.816	0.596	0.058	0.025
2	5	0.840	0.839	0.544	0.043	0.059	0.845	0.844	0.560	0.042	0.060
5	1	0.842	0.815	0.599	0.058	0.064	0.847	0.821	0.615	0.057	0.066
5	2	0.848	0.839	0.569	0.044	0.092	0.852	0.844	0.587	0.044	0.094
5	5	0.854	0.871	0.531	0.029	0.122	0.857	0.874	0.545	0.030	0.127
10	1	0.884	0.839	0.735	0.058	0.298	0.884	0.844	0.743	0.058	0.303
10	2	0.891	0.863	0.712	0.043	0.322	0.892	0.870	0.717	0.042	0.329
10	5	0.897	0.863	0.621	0.028	0.364	0.894	0.885	0.620	0.031	0.371

**Table 20:** Performance measures of SVR early integration (SO) for S2783 data set with reject option.

		**cv**					**test**				
***λ***	***α***	**Acc.**	**Prec.**	**Recall**	***FP *Rate**	**Reject**	**Acc.**	**Prec.**	**Recall**	***FP *Rate**	**Reject**
2	1	0.835	0.862	0.538	0.038	0.020	0.836	0.859	0.544	0.039	0.019
2	2	0.838	0.883	0.519	0.030	0.038	0.839	0.878	0.526	0.031	0.036
2	5	0.839	0.947	0.280	0.003	0.147	0.839	0.963	0.278	0.004	0.149
5	1	0.931	0.894	0.887	0.051	0.513	0.926	0.886	0.880	0.054	0.516
5	2	0.952	0.947	0.750	0.007	0.608	0.947	0.963	0.743	0.010	0.612
5	5	0.954	0.857	0.571	0.001	0.640	0.949	0.997	0.530	0.000	0.646
10	1	0.966	0.947	0.827	0.008	0.656	0.961	0.963	0.826	0.010	0.658
10	2	0.968	0.913	0.749	0.003	0.677	0.963	0.976	0.749	0.006	0.678
10	5	0.969	0.775	0.586	0.000	0.695	0.965	0.999	0.566	0.000	0.699

**Table 21:** Performance measures of SVM late integration for S2783 data set with reject option.

		**cv**					**test**				
***λ***	***α***	**Acc.**	**Prec.**	**Recall**	***FP *Rate**	**Reject**	**Acc.**	**Prec.**	**Recall**	***FP *Rate**	**Reject**
2	1	0.829	0.792	0.606	0.072	0.000	0.831	0.787	0.617	0.074	0.000
2	2	0.833	0.806	0.597	0.064	0.012	0.836	0.804	0.609	0.065	0.013
2	5	0.838	0.825	0.581	0.054	0.031	0.840	0.820	0.594	0.056	0.030
5	1	0.839	0.812	0.609	0.062	0.032	0.841	0.808	0.621	0.064	0.035
5	2	0.842	0.825	0.595	0.055	0.048	0.844	0.820	0.610	0.057	0.049
5	5	0.847	0.845	0.566	0.043	0.073	0.849	0.844	0.582	0.045	0.076
10	1	0.849	0.825	0.618	0.056	0.072	0.851	0.820	0.634	0.058	0.075
10	2	0.852	0.837	0.599	0.048	0.089	0.856	0.837	0.617	0.049	0.094
10	5	0.859	0.836	0.492	0.027	0.147	0.861	0.866	0.507	0.029	0.154

**Table 22:** Performance measures of SVR late integration for S2783 data set with reject option.

		**cv**					**test**				
***λ***	***α***	**Acc.**	**Prec.**	**Recall**	***FP *Rate**	**Reject**	**Acc.**	**Prec.**	**Recall**	***FP *Rate**	**Reject**
2	1	0.828	0.862	0.512	0.036	0.021	0.834	0.862	0.532	0.037	0.019
2	2	0.834	0.907	0.472	0.021	0.053	0.837	0.897	0.484	0.023	0.056
2	5	0.833	0.961	0.327	0.005	0.123	0.838	0.963	0.328	0.005	0.130
5	1	0.938	0.916	0.856	0.032	0.474	0.940	0.909	0.865	0.033	0.477
5	2	0.949	0.961	0.770	0.009	0.535	0.952	0.963	0.779	0.009	0.541
5	5	0.952	0.937	0.620	0.001	0.570	0.953	0.979	0.630	0.003	0.575
10	1	0.966	0.961	0.872	0.010	0.604	0.965	0.963	0.860	0.010	0.606
10	2	0.970	0.936	0.757	0.001	0.638	0.966	0.977	0.748	0.004	0.638
10	5	0.970	0.860	0.660	0.000	0.652	0.967	0.984	0.675	0.002	0.652

**Table 23:** Performance measures of SVM intermediate integration (TO*) for S2783 data set with reject option.

		**cv**					**test**				
***λ***	***α***	**Acc.**	**Prec.**	**Recall**	***FP *Rate**	**Reject**	**Acc.**	**Prec.**	**Recall**	***FP *Rate**	**Reject**
2	1	0.807	0.712	0.632	0.116	0.000	0.802	0.693	0.636	0.124	0.000
2	2	0.823	0.755	0.598	0.083	0.051	0.822	0.749	0.603	0.086	0.055
2	5	0.836	0.802	0.540	0.053	0.108	0.837	0.804	0.548	0.052	0.112
5	1	0.851	0.769	0.678	0.082	0.165	0.849	0.765	0.679	0.084	0.168
5	2	0.862	0.802	0.636	0.058	0.207	0.862	0.804	0.639	0.058	0.211
5	5	0.874	0.725	0.357	0.022	0.309	0.872	0.796	0.351	0.021	0.318
10	1	0.878	0.802	0.717	0.066	0.300	0.879	0.804	0.728	0.065	0.308
10	2	0.891	0.787	0.550	0.034	0.372	0.892	0.813	0.562	0.034	0.383
10	5	0.898	0.399	0.224	0.013	0.436	0.899	0.579	0.244	0.012	0.445

**Table 24:** Performance measures of SVR intermediate integration (TO) for S2783 data set with reject option.

		**cv**					**test**				
***λ***	***α***	**Acc.**	**Prec.**	**Recall**	***FP *Rate**	**Reject**	**Acc.**	**Prec.**	**Recall**	***FP *Rate**	**Reject**
2	1	0.810	0.723	0.595	0.099	0.035	0.808	0.715	0.596	0.102	0.037
2	2	0.839	0.843	0.405	0.026	0.177	0.840	0.855	0.393	0.024	0.185
2	5	0.843	0.589	0.101	0.001	0.258	0.841	0.946	0.103	0.003	0.260
5	1	0.918	0.887	0.629	0.021	0.505	0.917	0.889	0.615	0.022	0.515
5	2	0.926	0.589	0.275	0.002	0.555	0.924	0.946	0.268	0.004	0.562
5	5	0.926	0.310	0.127	0.000	0.565	0.925	0.877	0.125	0.001	0.573
10	1	0.961	0.589	0.436	0.003	0.698	0.952	0.946	0.458	0.005	0.697
10	2	0.963	0.310	0.200	0.000	0.708	0.953	0.877	0.256	0.001	0.708
10	5	0.963	0.310	0.200	0.000	0.708	0.953	0.877	0.256	0.001	0.708

## Discussion

We focus on the protein stability change prediction by adding new features and implementing the three different integration approaches, classification vs. regression, the effect of the reject option.

### Sufficiency of the Data Sets

**Figure 5:**
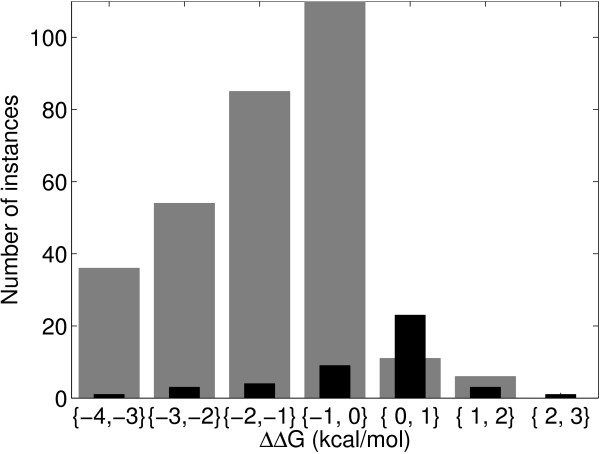
**Distribution of the correctly classified (grey) and misclassified (black) instances of S1615 data set after the SVM combiner over the free energy change due to single-site mutation, ΔΔ*G***. Misclassified instances are clustered mainly around zero. In the regions {-∞, -4} and {3, ∞} all instances are correctly classified.

Training any classifier with an unbalanced data set in favor of negative instances makes it difficult to learn the positive instances. The unbalanced distribution in prior probabilities of the two classes in both training and test sets affects the reliability of the predictor in all integration approaches. Nevertheless, the abundance of one class remains with the nature of the stability problem. Stabilizing mutations are far less than destabilizing mutations. Higher accuracies might be achieved with balanced training and test tests. For example, the test sets of S1615 and S2783 data sets have 88.51 per cent and 69.45 per cent destabilizing mutations, respectively. S1615 data set does not have balanced training and test sets whereas we evenly distribute stabilizing and destabilizing mutations to training and test sets for S2783 data set. For S1615 dataset, we achieve 0.904 the average test accuracy which is 1.90 per cent higher than the percentage of destabilizing mutations. For S2783 data set, this improvement is around 14.05 per cent. ΔΔ*G *values for the majority of both training and test data are in the interval {-1, 1}. We would expect the predictor to learn the pattern in this region better than the other regions in the data space. However, Figure 5 suggests that it is not the case, and this is in agreement with previous studies [9]. Even though the ΔΔ*G *values are not provided to the classification algorithm numerically, the error rate is higher for smaller changes and lower for larger ones. This may be due to two reasons: Either our predictor works best at dramatic stability changes; or possible experimental errors, being more significant for smaller ΔΔ*G *values than the larger ones, confused our predictor. In separating the mutations into two distinct classes as positive and negative, the prediction may be ambiguous for data points close to zero. If we test our best classifier for S1615 data set with the test instances outside of this interval (230 of 383 instances), we obtain 0.948 test accuracy. This last result shows the advantage of introducing a reject option and the approach we use by taking into account the losses of rejects and wrong decisions is the systematic way to choose the optimal thresholds.

Furthermore, the mutations in the test set of S1615 data set were conducted in physiological conditions [2], having T in the range 20-30°C and pH in the range 6-8 whereas for the training set, the ranges are 0-86°C and 1-11 respectively. It is not ideal to train a learner with data within a wide range and test it only in a limited region; it is normally expected that the training and test sets follow the same probability distribution. In S2783 data set, the test data and the training data are split randomly to alleviate this problem. Because we do the splitting ten times and take the average, our results are more robust on S2783 data set.

### Integration Approaches

The most accurate predictor in early integration for S1615 (S2783) data set is SVM (SVM) classifier trained with ST* (SO) achieving a validation set accuracy of 0.842 (0.829) and a test accuracy of 0.904 (0.832). We see in Tables 6 and 13 that using structural information is useful with *k*-NN and DT; adding new features such as PAM and CB improve cv accuracy, and in the case of TO*, also improves test accuracy using SVM, though not significantly. It may be said that TO does not have enough packing information intrinsically and using *B-factor*s and *C*_*β *_may help.

In late integration for S1615 data set, of the four triplets combined, two are SVM, one is DT and one is *k*-NN. The fact that four different learners are chosen show that the learning algorithm is a good source of diversity. Of the four, two use ST* and SO*, showing again that in terms of representations, there is also diversity for higher accuracy. Note that this diverse set is found automatically by the selection algorithm we use.

The most accurate intermediate integration version for S1615 data set uses TO* with all new features; its test accuracy is 0.879, which is significantly more accurate than the version with old features only (TO) with test accuracy 0.833. Though it is not as accurate as the other integration methods, intermediate integration has the advantage of knowledge extraction through weights assigned to features. The kernel weights (see Tables 11, 13, 17, and 18) show that when the protein structure is available, CA and CB are always preferred as a more valuable information source than any other features including sequence neighbors. Based on the kernel weights, we can say that stability change is mostly a structure-driven phenomenon: For example, when we sum up the weights of structural features for S1615 data set, using ST*, we get (0.18)CA + (0.09)SA + (0.26)CB + (0.08)BFA + (0.06)BFB = 0.67 of 1.00.

### Prediction Using Only the Amino Acid Sequence

We analyze simulation results to see how accuracy changes if we have only the sequence information. For both data sets, the best performance in early integration is obtained with (SO. ORIGINAL.SVM). The average test accuracies are 0.904 and 0.832 for S1615 and S2783 data sets, respectively. Intermediate integration for S1615 data set achieves 0.872 average testing accuracy with SO, which is higher than those of TO and ST (0.833 and 0.818, respectively). With the extra features, the accuracies are 0.872, 0.879, and 0.878 for SO*, TO*, and ST*, respectively (see Table 10). The improvement with additional information in TO* and ST* is not significant when compared with SO. For S2783 data set, intermediate integration achieves 0.800 test accuracy with SO. All feature representations achieve statistically similar test set accuracies for both multikernel SVM and SVR.

Prediction from only the sequence information could be considered more valuable at present as sequence-based data are more readily available. Even if the average accuracy is increased by extra structural features, these features are obtained through costly experimental procedures like x-ray crystallography or NMR spectroscopy. Spending more effort on making better use of sequence-only features with different learning methods might be more beneficial.

### Classification with Reject Option

When we compare the results of classification with reject option, we see that early and late integration methods tends to reject fewer test instances than intermediate integration with late rejects the least. For example, in order to achieve 0.850 test set accuracy, early and late integration need to reject around 10 per cent of the test instances whereas intermediate integration rejects around 15 per cent of the test instances (see Tables 19, 21, and 23). Another target can be achieving a specific *FP *rate. In this case, for example, early and late integration reject 10 per cent of the test instances and intermediate integration rejects 35 per cent of the test instances to get a *FP *rate less than 0.05. The same behavior can also be observed for regression (see Tables 20, 22, and 24).

### Comparison with Other Studies

Our methodology using 20-fold cv has comparable accuracy to previous studies [2,7,9,41]. S1615 data set is based on Protherm that has been also used by those studies. Nevertheless, it is not exactly the same data set as we remove the test set from the training set, thus we represent our comparison with this caveat in Table 25. Early integration approach is used in all referred works. They all report the performance of their predictors based on *k*-fold cv, also including the test set in cross-validation. The highest accuracy reported so far is 0.930 evaluated on a subset of the training data [9]; our early integration has the accuracy of 0.904 on the independent test set. In those studies, higher accuracies are reported in the presence of structural information, which is in agreement with our findings though the difference is not significant in our case. Ours is the first study that compares early, intermediate, and late integration to incorporate knowledge from different data sources for the problem of predicting protein stability, also analyzing the effect of different types of sequence and structural information.

**Table 25:** Comparison of our results for S1615 data set with previously published studies.

**Ref.**	**Method**	**Data Set Size**	**Accuracy**	**Information**
[41]	SVM	2048	0.77 (20-fold cv)	Seq

[42]	SVM	1383 *	0.73 (20-fold cv)	Seq

[9]	NN NN+FOLDX	1615	0.79 (20-fold cv) 0.87 (test set†) 0.93 (test set†)	Seq+Str

[2]	SVM	1496‡	SO: 0.84, TO: 0.85, ST: 0.85 (20-fold cv) SO: 0.86, TO: 0.86, ST: 0.86 (test set)	Seq+Str

[31]	iPTREE	1615	0.87 (10-fold cv)	Seq+Str

Ours	Early Late Intermediate	1122 (training) 383 (test)	**0.842 **(20-fold cv), **0.904 **(test set) 0.847 (20-fold cv), 0.903 (test set) 0.826 (20-fold cv), 0.879 (test set)	Seq+Str

*Filtered from the set of 2048 mutations [41].

† A subset of the training set that was previously used in training.

‡ Filtered from the set of 1615 mutations [9].

Machine learning method, data set, performance assessment are the main features to be compared. (Seq: Sequence-based information, Seq+Str: Sequence- and structure-based information)

## Conclusion

In protein stability prediction, we investigate three approaches for combining multiple representations/learners, namely, early, intermediate, and late integration. These approaches can be used in both classification and regression. Early integration uses a single learner to combine multiple inputs whereas late integration combines the decisions of learners using different inputs. Intermediate integration combines inputs at the kernel level. We find that early and late integration are significantly more accurate than intermediate integration and intermediate integration allows knowledge extraction in the sense that it can pinpoint the features that are useful and how much they contribute. One advantage of combination is that if a new feature set, a kernel or a method for learning is proposed (using machine learning or some other approach), it is always possible to include it among the set we use and thereby improve accuracy even further.

In general, we would expect early integration to suffer more from the curse of dimensionality when many input sources are concatenated. Late integration combines decisions and therefore is expected to be more robust; the disadvantage would be the need to train/store/use multiple learners. Intermediate integration is in between these two extremes where separate features are not used in a raw manner (as in early integration) nor are decisions extracted from them (as in late integration) but are converted to similarities (using kernels) and fed to a single learner. The relative weights of features can be measured using intermediate integration. Of course, ours is a single study and further research is needed before one can explain with enough confidence where and why each integration method works the best. Of the three which one should be chosen depends on the application and other criteria, such as how much time and space can be afforded.

We see that in terms of accuracy there is no significant difference between interpreting this as a classification or regression problem except that a regressor tends to have a lower *FP *rate. We also conclude that introducing a reject option is useful to reject cases where a classifier or a regressor is not confident; it allows achieving a much lower *FP *rate taking into account the loss incurred for rejections and misclassifications.

As a future direction, we can add features, for example, to reflect the side chain conformation change due to a single-site mutation by a simple modeling.

## Authors' contributions

AO and MG developed the concept and the method under the guidance of EA and TH. MG developed the web server. AO and MG drafted the paper. EA and TH finalized the draft. All authors read and approved the final manuscript.
